# A Novel Diagnostic Predictive Model for Idiopathic Short Stature in Children

**DOI:** 10.3389/fendo.2021.721812

**Published:** 2021-09-17

**Authors:** Jinghong Yuan, Zhi Du, Zhiwen Wu, Yanqin Yang, Xigao Cheng, Xijuan Liu, Jingyu Jia

**Affiliations:** ^1^Department of Orthopaedics, The Second Affiliated Hospital of Nanchang University, Nanchang, China; ^2^Department of Pediatrics, The Second Affiliated Hospital of Nanchang University, Nanchang, China

**Keywords:** idiopathic short status, C1QA, C1QB, inflammation, mass spectrometry—LC-MS/MS, diagnosis, endochondral ossification

## Abstract

**Objective:**

Idiopathic short stature (ISS), an endocrine-related disease, is difficult to diagnose. Previous studies have shown that many children with some inflammation-related diseases often have short stature, but whether inflammation is the underlying mechanism of ISS has not been studied. Here, we attempt to explore the role of inflammation in the occurrence and development of ISS and to demonstrate an available clinical diagnostic model of ISS.

**Methods:**

Frozen serum samples were collected from ISS patients (n = 4) and control individuals (n = 4). Isobaric tags for relative and absolute quantitation (iTRAQ) combined with LC-MS/MS analysis were applied to quantitative proteomics analysis. To assess clusters of potentially interacting proteins, functional enrichment (GO and KEGG) and protein-protein interaction network analyses were performed, and the crucial proteins were detected by Molecular Complex Detection (MCODE). Furthermore, serum levels of two selected proteins were measured by ELISA between ISS patients (n = 80) and controls (n = 80). In addition, experiments *in vitro* were used to further explore the effects of crucial proteins on endochondral ossification.

**Results:**

A total of 437 proteins were quantified, and 84 DEPs (60 upregulated and 24 downregulated) were identified between patients with ISS and controls. Functional enrichment analysis showed that the DEPs were primarily enriched in blood microparticle, acute inflammatory response, protein activation cascade, collagen-containing extracellular matrix, platelet degranulation, etc. According to the results of top 10 fold change DEPs and MCODE analysis, C1QA and C1QB were selected to further experiment. The expression levels of C1QA and C1QB were validated in serum samples. Based on the logistic regression analysis and ROC curve analysis, we constructed a novel diagnostic model by serum levels of C1QA and C1QB with a specificity of 91.2% and a sensitivity of 75% (AUC = 0.900, p <0.001). Finally, the western blotting analysis confirmed the expression levels of OCN, OPN, RUNX2, and Collagen X were downregulated in chondrocytes, and the outcome of Collagen II was upregulated.

**Conclusion:**

Our study is the first to demonstrate the significant role of inflammation in the development of ISS. In addition, we identify C1QA and C1QB as novel serum biomarkers for the diagnosis of ISS.

## Introduction

The previous definition of idiopathic short stature (ISS) was that in children with normal body proportions, standard birth size, and without any systemic, nutritional, endocrine, or chromosomal abnormalities, there is presence of a height more than two standard deviations (SD) below the corresponding average size for a given age, gender, and population (1). Currently, the diagnosis of ISS is complicated because other identifiable causes of short stature must be ruled out (2), and the primary treatment of ISS is to inject recombinant human growth hormone into children with ISS; however, the effect of this treatment is not satisfactory (3). The reason for these difficulties was the unclear pathogenesis of ISS. Therefore, the pathogenesis of ISS needs to be clarified to discover a novel accurate diagnosis method and to improve the treatment method of ISS.

Children with chronic inflammatory diseases, such as inflammatory bowel disease (IBD), cystic fibrosis (CF), juvenile idiopathic arthritis (JIA), and intrauterine growth restriction (IUGR) were often accompanied by growth and development disorders (4–8). Up to 19–31% of children with IBD have a developmental delay as the initial clinical manifestation (9–11), and the research of Kirschner suggested that children with IBD have a potential permanent effect on inhibiting linear growth (12). Lai et al. reported that 20% of patients with CF also have short stature (5). The research of Susanne et al. showed that 30–40% of children with JIA have generalized or multiple joint JIA, and these patients with JIA have a higher incidence of growth disorders (6). However, few studies have shown that idiopathic short stature is related to inflammation (13). Therefore, we suspect that inflammation may be the pathogenesis of ISS.

Short stature is a complex multifactorial disease affected by genetic, epigenetic, and environmental factors (14, 15). Philip et al. reported that 25–40% of patients diagnosed with ISS could be diagnosed by some molecular genetic techniques, such as copy number variant (CNV) analysis, the single-gene approach, and whole-exome sequencing (WES) (14). Specific genetic defects were confirmed to be related to ISS, including aggrecan (ACAN), SHOX, fibroblast growth factor receptor 3 (FGFR3), components of the GH/IGF-I axis, and natriuretic peptide receptor 2 (NPR2) (15).

In this research, quantitative proteomics, such as liquid chromatography tandem mass spectrometry (LC-MS/MS), has been a dominant method to identify the protein profiling of serum and discover the novel potential diagnostic biomarkers of most diseases in serum (16–18). As we all know, ISS is correlated to the endocrine (2). Therefore, serum samples could provide much information to study ISS for us. Serum biochemical analysis may be used as a more economical and convenient method for the diagnosis of ISS.

In the present study, isobaric tags for relative and absolute quantitation (iTRAQ) combined with LC-MS/MS analysis were firstly applied to identify potential pathogenesis and serum biomarkers between patients with ISS and controls. Differential expressed proteins (DEPs) in serum were mainly related to immune inflammation biological processes. The crucial serum DEPs (C1QA and C1QB) were verified using enzyme-linked immunosorbent assay (ELISA), and their expression levels were higher than the controls. Our results showed for the first time that a diagnostic model composed of C1QA and C1QB, based on logistic regression, could powerfully diagnose ISS more accurately and conveniently. Furthermore, experiments *in vitro* confirmed the effects of C1QA and C1QB on endochondral ossification of chondrocyte for the first time.

## Materials and Methods

### Patients

From May 2019 to August 2020, a total of 168 blood samples from 168 patients with idiopathic short stature (ISS) and age-/sex-matched control individuals were enrolled in the present study, and these samples were collected from the Second Affiliated Hospital of Nanchang University (Nanchang, China). The conditions for patient inclusion in this study are consistent with our previous study (19). All of the blood samples were immediately frozen in liquid nitrogen and stored at −80°C until further experiment. Four pairs of blood samples were used for iTRAQ and LC-MS/MS analysis. For the validation set, another eighty pairs of samples were collected.

### Protein Preparing and iTRAQ Labeling

Patients with ISS and control individuals were randomly divided into two groups: ISS1–ISS4 for the patients with ISS and CON1–CON4 for the control specimens. After obtaining the same amount of serum from all of the samples, Pierce™ Top 12 Abundant Protein Depletion Spin Columns (ThermoScientific Biosciences, 85165) was applied to remove the top 12 high abundance serum proteins according to the instructions. Protein concentration was identified using a BCA assay (ThermoScientific Biosciences, 23227). For each sample, proteins (10 μg) were separated by 12% SDS-PAGE gel. The gel was stained with 0.2% Coomassie Brilliant Blue G-250 (CBB, Sigma, 27815-25G-F) according to Candiano’s protocol (20). After the stained gel was washed with water until bands were displayed, the gel was scanned with ImageScanner (GE Healthcare, USA) at the resolution of 300 dpi. According to the manuscript instructions, the proteins underwent trypsin digestion, and the digested peptides were collected. The peptides were labeled with regents following the iTRAQ kit (ABSCIEX, 4381663) instructions for further analysis.

### LC-MS/MS

An 1100 HPLC System (Agilent) was applied for reversed-phase liquid chromatography (RPLC) using the following parameters: Angilent ZORBAX Extend-C18 column (2.1 × 150 mm, 5 μm); separated at a flow rate of 300 μl/min; monitored at 210 and 280 nm UV detection. According to the instructions, the peptides were collected at 1 min intervals for the 8th–60th minutes. After recycling in this order until the end of the gradient, a total of 15 segments were lyophilized for mass spectrometry analysis. All of the data acquisition and analyses were performed by a Triple TOF 5600 mass spectrometer (SCIEX, USA) equipped with a Nanospray III source (SCIEX, USA) and Proteome Discover 2.3 (ThermoScientific Biosciences).

### Protein Identification and Potential Interactions Cluster Assessment

The protein screening criteria were as follows: unused >1.3 and unique peptide ≥1. Differentially expressed proteins (DEPs) screening criteria were as follows: fold-change (FC) ≥ 1.2 or ≤ 0.83 and P-value < 0.05. The heat map of DEPs was constructed using hierarchical clustering, which was performed using the 3.12 version of ComplexHeatmap package (http://www.bioconductor.org/pac-kages/release/bioc/html/ComplexHeatmap.html) (21) in R 3.6.0 software (https://www. R-project.org/). Based on the annotation information from Uniprot database, the functional enrichment analyses on the DEPs from the mass spectrometry analysis were carried out using Metascape (https://metascape.org), a user-friendly online analysis tool for gene annotation and analysis. Only terms with P-value less than 0.01, minimum count of 3, and enrichment factor more than 1.5 were screened. In this study, functional enrichment analyses included Gene Ontology (GO: https://www.geneontology.org) and Kyoto Encyclopedia of Genes and Genomes (KEGG: https://www.genome.jp/kegg/). GO is a major bioinformatics project, including biological processes (BP), molecular function (MF), and cellular component (CC). KEGG pathway enrichment analysis of DEPs was able to determine the enriched pathways, which is essential for identifying significant biological regulatory pathways. A protein-protein interaction (PPI) network of DEPs was constructed, and the crucial proteins in this PPI network were screened using the Molecular Complex Detection (MCODE, http://apps.cytoscape.org/apps/mcode), a plug-in of Cytoscape (version 3.7.2, https://cytoscape.org/). All the analysis was performed on the Metascape website (22) using the following parameters: Min Network Size = 3 and Max Net-work Size = 500. In addition, crucial proteins (Hub Genes) in the MCODE networks were detected and visualized by comparing the top 10 DEPs using ggplot2 package (https://cran.r-project.org/web/packages/ggplot2/index.html) in R.

### Enzyme-Linked Immunosorbent Assay

Patients with ISS (80 cases) and controls (80 cases) were indicated in the ELISA verification cohorts. C1QA and C1QB were selected as crucial biomarkers for further analysis in the verification cohorts. According to the manufacturer’s instructions, serum levels of C1QA was measured using the ELISA Kit (SED207Hu, Cloud-Clone Corp, Wuhan, China), and the serum levels of C1QB was measured using the ELISA Kit (SED208Hu, Cloud-Clone Corp, Wuhan, China). In this study, a microplate reader (Varioskan Flash 2.4, Thermo Fisher Scientific) was used to determine the OD value of each well at 450 nm.

### Culture and Stimulation of Chondrocytes

Human chondrocytes (C28) were purchased from Procell (Wuhan, China) and cultured in Dulbecco’s modified Eagle’s medium (Gibco, DMEM) with 10% FBS (Gibco, South America), and maintained in a 37°C environment with 5% CO2. After seeding the chondrocytes (1 × 105/well) in six-well plates for 12 h, the chondrocytes were stimulated for 24 h with C1QA (10 mg/ml, APD207Hu01, Cloud-Clone Corp, Wuhan, China), C1QB (10 mg/ml, APD208Hu01, Cloud-Clone Corp, Wuhan, China), or mix of C1QA (10 mg/ml, APD207Hu01, Cloud-Clone Corp, Wuhan, China) and C1QB (10 mg/ml, APD208Hu01, Cloud-Clone Corp, Wuhan, China) for further experiment.

### Western Blot Analysis

The proteins were extracted from stimulated and control chondrocytes by using the RIPA lysis buffer (cat. no. P0013B, Beyotime Institute of Biotechnology), and concentration was measured using a BCA protein assay kit (Pierce Biotechnology Inc., Rockford, IL, USA). Then 10 μg/lane protein samples were separated by 10% SDS-PAGE, and the separated proteins were transferred to a 0.2 μM polyvinylidene difluoride (PVDF) membrane (Bio-Rad). After blocking in 5% non-fat dry milk in TBS-T (0.1% Tween 20) for 2 h at 37°C, the PVDF membrane was incubated with anti-OCN (4°C; 8 h; 1:1,000; cat. no. ab133612), anti-OPN (4°C; 8 h; 1:1,000; cat. no. ab214050), anti-C1QA (4°C; 8 h; 1:1,000; cat. no. ab189922), anti-C1QB (4°C; 8 h; 1:1,000; cat. no. ab92508), anti-RUNX2 (4°C; 8 h; 1:5,000; cat. no. Ab192256), Collagen X (4°C; 8 h; 1:1,000; cat. no. ab58632), Collagen II (4°C; 8 h; 1:1,000; cat. no. ab188570), and GAPDH (4°C; 8 h; 1:5,000; cat. no. Ab9485). After washing with TBS-T (0.1% Tween 20) for 10 min three times, the PVDF membrane was incubated with goat anti-rabbit IgG antibodies (37°C; 2 h; 1:5,000; cat. no. ab80673). Finally, the membrane was washed, and the detection was performed using Super ECL Plus (Cat: S6009, US EVERBRIGHT). The results of western blotting were analyzed using the Tanon 4600 gel image analysis software (version 4.2, Tanon, Shanghai, China).

### Statistical Analysis

In the present study, logistic regression analysis and areas under curve (AUC) of receiver operating characteristic (ROC) analysis were used to evaluate the ability of C1QA and C1QB on diagnosis of ISS by the 1.17.0.1 version of pROC package (https://cran.r-project.org/web/packages/pROC/index.html) and visualized by ggplot2 package (https://cran.r-project.org/web/packages/ggplot2/index.html) in R. The differences in clinical data outcomes of experiments were evaluated using Mann-Whitney U test, Pearson’s chi-squared test, and Student’s t-test, and the mean ± standard deviation (SD) was used to assess the continuous variables. P-values less than 0.05 were determined to be statistically significant.

## Results

### Screening of DEPs and Functional Enrichment Analysis

**Figure 1:**
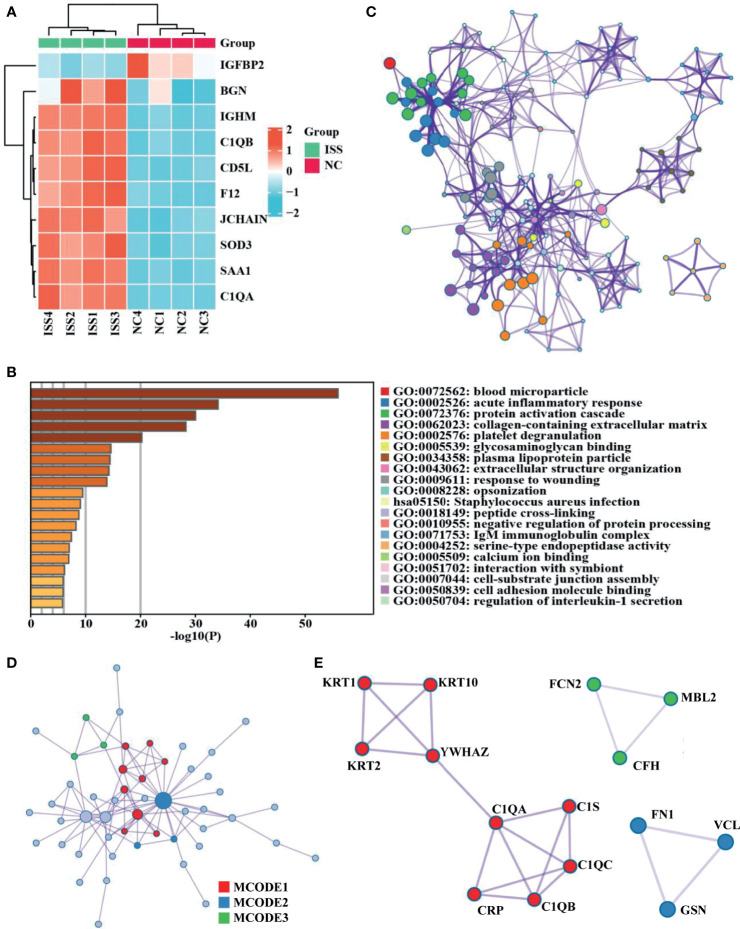
Differential expression proteins analysis and crucial proteins selected. **(A)** A clustering heat map of the top 10 fold change for differentially expressed proteins (DEPs) patterns of ISS and negative control (NC) groups. Red color indicates higher expression, while blue indicates lower expression. Proteins without significantly differential expression are shown in white. **(B)** Significant functional enrichment analysis results of DEPS *via* Metascape database. **(C)** Enrichment network plot of DEPS constructed by Cytoscape software. **(D, E)** Results of MCODE’s subnetwork analysis of the PPI network; three key subnetworks were obtained, namely, MCODE 1 (red), MCODE 2 (blue), and MCODE 3 (green).

A total of 437 proteins were quantified, and 84 DEPs (60 upregulated and 24 downregulated) were identified between patients with ISS and controls (**Supplementary Table 1**), and the top 10 FC (Fold Change) for DEPs were visualized by a heat map (**Figure 1A**). To determine the function of these 84 DEPs in ISS, Metascape was used for functional enrichment analysis. The results showed as a bar graph that the DEPs were primarily enriched in blood microparticle, acute inflammatory response, protein activation cascade, collagen-containing extracellular matrix, platelet degranulation, etc. (**Figure 1B** and **Supplementary Table 2**). To analyze the intercluster similarities and intracluster redundancies, an enrichment network plot was constructed using Cytoscape software (**Figure 1C**). In the network, each enriched term was represented as a node, and edges linked these terms (Kappa similarity > 0.3). Based on the results of GO and KEGG pathway enrichment analyses on the Metascape website, the three most crucial enriched terms were used to determine putative biological roles for each MCODE complex and visualized in **Figures 1D, E**, namely, MCODE_1 (Staphylococcus aureus infection, complement activation, and protein activation cascade; C1QA, C1QB, C1QC, C1S, CRP, KRT1, KRT2, KRT10, and YWHAZ), MCODE_2 (Regulation of actin cytoskeleton, secretory granule lumen, and cytoplasmic vesicle lumen; FN1, VCL, and GSN), and MCODE_3 (complement activation, protein activation cascade, and humoral immune response; FCN2, MBL2, and CFH).

### Verification of DEPs and Clinical Characteristic

**Figure 2:**
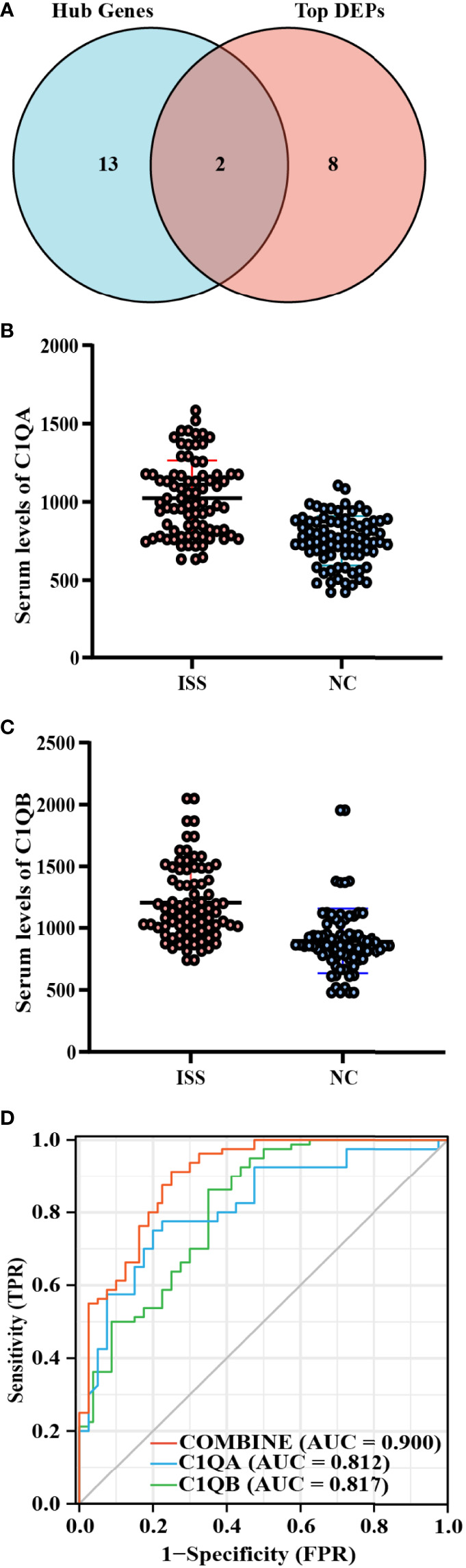
Diagnostic model construction of ISS. **(A)** Venn diagram of intersected targets of Hub genes and the top 10 FC DEPs. The serum expression levels of C1QA **(B)** and C1QB **(C)** between ISS and control groups, FC = XXX, P < 0.05. **(D)** Receiver operating characteristic (ROC) for predictive values of C1QA (blue), C1QB (green), and combined (red) levels in detecting ISS.

C1QA and C1QB were common between the top 10 FC DEPs and hub genes from MCODE networks (**Figure 2A**, **Supplementary Table 3**). Based on the functional enrich analysis of the LC-MS/MS results, the serum levels of C1QA and C1QB were significantly increased in patients with ISS compared with controls, and the crucial enriched terms were related to immunity and inflammation, including acute inflammatory response, Staphylococcus aureus infection, complement activation, humoral immune response, IgM immunoglobulin complex, etc. Therefore, we assumed that inflammation and immune-related factors such as C1QA and C1qB may play an essential role in the potential pathogenesis of ISS. ELISA assay was used to determine the serum levels of C1QA and C1QB in another cohort of 160 patients (80 patients with ISS and 80 control patients).

Comparing the differences in clinical characteristics and laboratory experiments between patients with ISS and the controls, the results suggested that the variables of WBC (White blood cell), Height, Weight, CRP (C-reactive protein), and chronological age were significantly statistically different between the two groups (**Table 1**). On the basis of ELISA assay, the serum levels of C1QA (FC = 1.36, P < 0.001) and C1QB (FC = 1.34, P < 0.001) were significantly statistically different between ISS and control groups (**Figures 2B, C**).

**Table 1:** Comparison of patients with ISS and normal control individuals (NC).

Group	ISS	Controls	P-value
**No. children**	80	80	–
**Gender (male/female)**	41/39	40/40	0.87
**Age, y**	9.68 ± 2.50 (5–14)	9.59 ± 2.01 (5–14)	0.61
**Chronological age, y**	5.89 ± 1.93 (3–11)	9.41 ± 2.09(5–15)	<0.001
**Height, cm**	122.17 ± 14.03 (85.5–148.7)	138.41 ± 10.42 (117.8–159)	<0.001
**Weight, kg**	23.46 ± 6.42 (13.2–39.1)	33.79 ± 6.76 (23.4–53.2)	<0.001
**WBC, 10^9^/L**	7.91 ± 2.00 (3.98–15.55)	7.38 ± 2.10 (3.96–15.21)	0.049
**CRP, mg/L**	6.26 ± 3.43 (0.13–13.85)	0.70 ± 0.97 (0.08–6.32)	<0.001

### ROC Curve Analysis

After constructing the ROC curves for biomarkers, the results showed that the expression levels of C1QA and C1QB were significantly different between ISS and control groups (**Figure 2D**). The ROC curve showed that the serum levels of C1QA and C1QB predicted the status of primary gout with middle accuracy (C1QA: AUC = 0.812, p < 0.001; C1QB: AUC = 0.817, p < 0.001; **Supplementary Table 4**). The C1QA with the best cut-off point of 899.33 ng/ml has a specificity of 86.2% and a sensitivity of 65.0%. Furthermore, the C1QB with the best cut-off point of 941.655 ng/ml has a specificity of 75.0% and a sensitivity of 80.0%. To find more well-balanced biomarkers for the diagnosis of ISS, a diagnostic model was established using logistic regression analysis about C1QA and C1QB. In the present study, the coefficients were as follows: −12.612 + 0.009*C1QA + 0.005*C1QB. The novel diagnostic model had a specificity of 91.2% and a sensitivity of 75% (AUC = 0.900, p < 0.001; **Figure 2D**).

### Increased C1QA and C1QB Reduce Chondrocyte Osteogenesis

**Figure 3:**
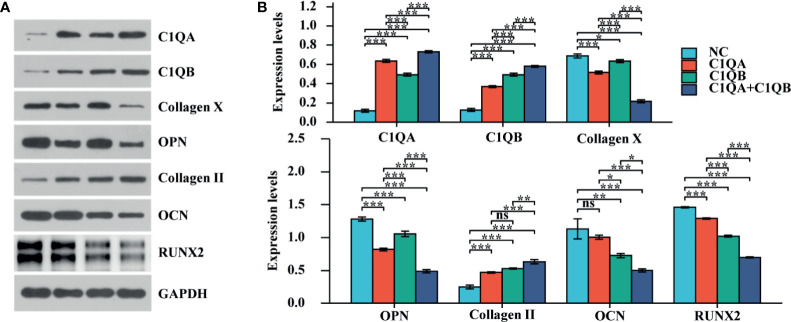
The effect of C1QA and C1QB on chondrocyte mineralization. **(A)** Western blotting results of the osteogenesis-related proteins (OCN, OPN, RUNX2, Collagen X, and Collagen II). **(B)** The densitometric analysis of western blotting, *P <0.05, **P < 0.01, ***P < 0.001; ns, not significant.

To identify the effect of C1QA and C1QB on chondrocyte mineralization, experiments were used *in vitro*. The results of the osteogenesis-related proteins (OCN, OPN, RUNX2, Collagen X, and Collagen II) were quantified by using western blotting analysis. The expression levels of OCN, OPN, RUNX2, and Collagen X were downregulated in chondrocytes, and the outcome of Collagen II was upregulated (**Figures 3A, B**; *P < 0.05, **P < 0.01, ***P < 0.001).

## Discussion

Although the pathogenesis, diagnosis, and treatment of ISS have been researched in recent years, the exact etiology of ISS remains unclear and diagnosis of ISS is also complicated and difficult (1, 2). Currently, most studies suggested that the pathogenesis of ISS is pretty complicated, including endocrine, genetic, nutritional deficiencies, etc. (2–8, 14, 15). Here, we are the first to propose that inflammation is a potential pathogenesis of ISS and establish a novel clinical diagnostic model based on the expression levels of C1QA and C1QB.

In the present study, we firstly determined the 84 DEPs using the proteomic analysis in serum samples from patients with ISS and controls. By using enrichment analysis, PPI network construction, and MCODE screening, the results showed that C1QA and C1QB may be able to contribute to the pathogenesis of ISS by participating in the biological process of inflammation. After ELISA assay, it was confirmed that C1QA and C1QB expressions of children with ISS were higher than normal control samples. C1QA combined with C1QB could effectively diagnose ISS with a specificity of 91.2% and a sensitivity of 75% (AUC = 0.900, p < 0.001). In addition, an interesting finding in our outcomes of this research was that WBC and CRP were in normal range in both groups of ISS and NC, but WBC and CRP were relatively higher in the ISS group than the NC group. Furthermore, the results of cell experiment firstly suggested that C1QA and C1QB inhibited the endochondral ossification of chondrocyte, ultimately leading to ISS.

C1QA is an essential component of C1Q, a complement protein. The research of Lin et al. suggested that the expression of C1QA in the mouse retina activated the complement system in the mouse retina and induced an inflammatory response in the retina based on the injection of exogenous amyloid β1-42 (Aβ1-42) (23). Natalie et al. found that C1QA expression in patients with tuberculosis was higher than normal, and the increased C1QA expression was correlated with a dosage effect of the G allele using TaqMan^®^ SNP (single-nucleotide polymorphisms) assays and ELISA (24). The experiment results of Wang et al. showed that high expression levels of C1QA upregulated RIG-I-mediated activation of nuclear factor-κB, IFN-stimulated response element (ISRE), and transcription of IFN-β in 293T cells, and played a significant role in the innate immune response (25). In the current research, the expression level of C1QA was upregulated in the serum of patients with ISS.

C1QB is also an important part of the complement C1Q. The studies of Wang et al. and Lee et al. showed that C1QB was upregulated in tibialis anterior muscles of amyotrophic sclerosis (ALS) mouse and increased the disease progression through complement induction verification (26, 27). In the research of Byrnes, adult male rats were used to construct model of spinal cord injury (SCI), and found that inflammation-related genes were long-term upregulated in the SCI model by using microarray analysis and protein expression (28). Our results indicated that C1QA protein expression in ISS group is higher than the normal group.

As mentioned before, many inflammation-related diseases (IBD, CF, JIA, and IUGR) are closely related to the occurrence of short stature. However, the exact potential mechanisms of inflammation in the development of short stature remain unclear. In the current research, we are the first to propose and find that inflammation plays a major role in the development of ISS, and construct an excellent clinical diagnostic model of ISS according to the combined expression of C1QA and C1QB in the serum. The western blotting outcomes of osteogenesis-related proteins suggest that inflammation may cause ISS by inhibiting chondrocytes to form osteogenesis. In further studies, we should validate this diagnostic model in clinical trials, including prospective and multiple-center studies. And we could use more molecular biology techniques to further examine the molecular mechanisms of the two biomarkers in ISS.

The onset of ISS is usually related to a complex genetic background. A series of research has determined that the pathogenesis of ISS was related to ACAN (29, 30), GHR (31, 32), NPR2 (33), CYP26C1 (34), AKNRD11 (35), SHOX, and IGFALS (36) genes. To study the role of C1QA and C1QB in the complex genetic background of ISS, we have performed whole-exome sequencing on the four ISS samples used for protein profiling in this study at the same time. By literature search and whole-exome sequencing results, we have not been able to obtain accurate correlation between C1QA and C1QB and genetic background. In future researches, we will expand the sample size for whole-exome sequencing to further study the role of C1QA and C1QB in the genetic background of ISS.

In conclusion, the present study was the first to identify C1QA and C1QB as novel serum biomarkers model, based on logistic regression, for the diagnosis of ISS. Moreover, C1QA and C1QB synergistically inhibit mineralization in chondrocytes.

## Data Availability Statement

The original contributions presented in the study are included in the article/**Supplementary Material**. Further inquiries can be directed to the corresponding authors.

## Ethics Statement

The studies involving human participants were reviewed and approved by the Ethics Committee of the Second Affiliated Hospital of Nanchang University. Written informed consent to participate in this study was provided by the participants’ legal guardian/next of kin.

## Author Contributions

JY conceived the research, designed the analytical strategies, collected the clinical samples, performed data analyses, performed the experiments, and wrote the manuscript. ZD performed data analyses and wrote the manuscript. ZW collected the clinical samples and performed the experiments. YY and XC performed data analyses. XL and JJ conceived the research and wrote the manuscript. All authors contributed to the article and approved the submitted version.

## Funding

This research was supported by the National Nature Science Foundation of China (grant nos. 81960392 and 82060169), the Project of Science and Technology Department of Jiangxi Province (grant no. 20202BABL206038), and J and Jiangxi Provincial Department of Education (grant nos. GJJ200109 and GJJ200120).

## Conflict of Interest

The authors declare that the research was conducted in the absence of any commercial or financial relationships that could be construed as a potential conflict of interest.

## Publisher’s Note

All claims expressed in this article are solely those of the authors and do not necessarily represent those of their affiliated organizations, or those of the publisher, the editors and the reviewers. Any product that may be evaluated in this article, or claim that may be made by its manufacturer, is not guaranteed or endorsed by the publisher.
