# Supplementary material for: A Novel Diagnostic Predictive Model for Idiopathic Short Stature in Children
Source: Front Endocrinol (Lausanne). 2021 Sep 17;12:721812. doi: 10.3389/fendo.2021.721812 (PMC8485046; doi:10.3389/fendo.2021.721812)
Supplement: Supplementary file 1 [file Table_1.docx]

**Supplementary Table 1.** Differential expressed proteins between patients with ISS and normal controls.

| **Gene name** | **P-value** | **FC** |
| --- | --- | --- |
| IGHM | 0.00000015 | 4.229411765 |
| SOD3 | 0.00000945 | 3.770423375 |
| CD5L | 0.00000858 | 2.969727047 |
| SAA1 | 0.00000025 | 2.787878788 |
| IGFBP2 | 0.04440646 | 0.431305903 |
| C1QA | 0.00000157 | 2.288121661 |
| F12 | 0.00001512 | 2.201280512 |
| BGN | 0.01477349 | 2.165413534 |
| C1QB | 0.00000238 | 2.163305654 |
| JCHAIN | 0.00000426 | 1.897500905 |
| C4BPB | 0.00000229 | 1.866356145 |
| FN1 | 0.00001678 | 1.838892832 |
| THBS4 | 0.00426069 | 1.787108014 |
| ORM1 | 0.00000099 | 0.56128025 |
| C4BPA | 0.00000012 | 1.753872633 |
| HPR | 0.00000194 | 0.571091909 |
| C1QC | 0.00000551 | 1.707275804 |
| ITGA2B | 0.00076174 | 1.691453567 |
| IGHA1 | 0.00003280 | 1.678834561 |
| CRP | 0.00002337 | 1.640594059 |
| LPA | 0.00000324 | 1.638522427 |
| ACTN1 | 0.00010803 | 1.62295082 |
| DCD | 0.00000814 | 0.618122977 |
| ORM2 | 0.00000050 | 0.638001638 |
| CRISP3 | 0.00000100 | 0.649824706 |
| FCN2 | 0.00009730 | 1.518098835 |
| THBS1 | 0.00000501 | 1.510825227 |
| PZP | 0.00495944 | 0.662855955 |
| ALB | 0.00000010 | 0.672731075 |
| IGHG4 | 0.00014877 | 1.482159479 |
| IGHG1 | 0.00000078 | 0.676378118 |
| APOA4 | 0.00000000 | 1.474335189 |
| MBL2 | 0.00009762 | 0.687763713 |
| CETP | 0.00124878 | 1.452038002 |
| CD93 | 0.00571032 | 1.421307506 |
| APOC3 | 0.00001063 | 0.703940362 |
| KRT10 | 0.00000125 | 0.704453441 |
| A2M | 0.00000000 | 1.400240024 |
| CRTAC1 | 0.00563262 | 1.371775867 |
| CILP2 | 0.00062545 | 1.37037037 |
| HP | 0.00001141 | 0.737836664 |
| APMAP | 0.00050056 | 0.743679163 |
| PLTP | 0.00067391 | 1.342898975 |
| PI16 | 0.00003541 | 1.335474453 |
| IGHD | 0.01620081 | 0.754770783 |
| SERPINA3 | 0.00000002 | 0.761726492 |
| SELL | 0.00003294 | 0.763447972 |
| VCL | 0.00025876 | 1.302158273 |
| TNXB | 0.00000539 | 1.297242964 |
| SERPINA5 | 0.00073991 | 1.295924225 |
| TAGLN2 | 0.02299331 | 1.29226361 |
| MEGF8 | 0.04680902 | 1.291977077 |
| YWHAZ | 0.03005802 | 1.282087849 |
| AHSG | 0.00000833 | 1.277255907 |
| PCYOX1 | 0.01693002 | 1.269863791 |
| MCAM | 0.00157229 | 1.269503546 |
| MMP9 | 0.02688578 | 1.267933088 |
| HRG | 0.00018598 | 1.265005663 |
| KRT2 | 0.00002703 | 0.79153605 |
| COL6A3 | 0.00303636 | 1.25960452 |
| LCN2 | 0.03552821 | 1.24943757 |
| GPX3 | 0.00042714 | 0.80243298 |
| IGHV3-15 | 0.01396866 | 1.245018243 |
| ENO1 | 0.00742171 | 1.244388328 |
| CTSD | 0.00251472 | 0.803833145 |
| CKM | 0.01981009 | 1.241456583 |
| IGKC | 0.00009950 | 1.23929471 |
| C1S | 0.00246732 | 1.238668159 |
| IGKV3-20 | 0.02234731 | 1.23838836 |
| PROZ | 0.01801827 | 1.237136465 |
| CPB2 | 0.00029395 | 1.233389168 |
| APOA1 | 0.00000211 | 1.227791701 |
| KRT1 | 0.00025378 | 0.814882033 |
| CDH13 | 0.00160237 | 1.225932109 |
| HLA-A | 0.03274501 | 1.22098834 |
| AOC3 | 0.00644002 | 1.220033296 |
| PROCR | 0.01419015 | 1.219478357 |
| SERPINA4 | 0.00118960 | 0.824401368 |
| GC | 0.00003555 | 0.824817518 |
| CFHR1 | 0.00129407 | 1.205955335 |
| NRP1 | 0.00961979 | 1.203856749 |
| CFH | 0.00034585 | 1.203581267 |
| GSN | 0.00000144 | 1.2035252 |
| SERPINF2 | 0.00026720 | 0.831730769 |
