# Supplementary material for: A Novel Diagnostic Predictive Model for Idiopathic Short Stature in Children
Source: Front Endocrinol (Lausanne). 2021 Sep 17;12:721812. doi: 10.3389/fendo.2021.721812 (PMC8485046; doi:10.3389/fendo.2021.721812)
Supplement: Supplementary file 2 [file Table_2.docx]

**Supplementary Table 2.** DEPs functional enrichment analysis.

| **Category** | **GO** | **Description** | **Hits** | **Enrichment** | **Z-score** | **Log**  **(q-value)** |
| --- | --- | --- | --- | --- | --- | --- |
| GO CC | GO:0072562 | blood microparticle | A2M\|SERPINA3\|AHSG\|ALB\|APOA1\|APOA4\|C1QB\|C1QC\|C1S\|C4BPA\|CD5L\|FCN2\|FN1\|GC\|GSN\|CFH\|CFHR1\|HP\|HPR\|HRG\|IGHA1\|IGHD\|IGHG1\|IGHG4\|IGHM\|JCHAIN\|IGKC\|ITGA2B\|KRT1\|ORM1\|ORM2\|SERPINF2\|PZP\|YWHAZ\|IGKV3-20 | 69 | 49 | -52 |
| GO BP | GO:0002526 | acute inflammatory response | A2M\|SERPINA3\|AHSG\|C1QA\|C1QB\|C1QC\|C1S\|C4BPA\|C4BPB\|CD5L\|CPB2\|CRP\|F12\|FN1\|CFH\|CFHR1\|HP\|HPR\|IGHG1\|IGHG4\|IGKC\|MBL2\|ORM1\|ORM2\|SERPINF2\|SAA1\|IGKV3-20 | 35 | 30 | -30 |
| GO BP | GO:0072376 | protein activation cascade | A2M\|C1QA\|C1QB\|C1QC\|C1S\|C4BPA\|C4BPB\|CD5L\|CPB2\|CRP\|F12\|FCN2\|CFH\|CFHR1\|IGHA1\|IGHD\|IGHG1\|IGHG4\|IGHM\|IGKC\|KRT1\|MBL2\|IGHV3-15\|IGKV3-20 | 35 | 28 | -26 |
| GO CC | GO:0062023 | collagen-containing extracellular matrix | A2M\|SERPINA3\|AHSG\|APOA1\|APOA4\|APOC3\|BGN\|C1QA\|C1QB\|C1QC\|CDH13\|COL6A3\|CTSD\|F12\|FN1\|HRG\|KRT1\|MBL2\|MMP9\|ORM1\|ORM2\|SERPINA5\|SERPINF2\|PZP\|SOD3\|THBS1\|THBS4\|TNXB | 20 | 23 | -25 |
| GO BP | GO:0002576 | platelet degranulation | A2M\|SERPINA3\|ACTN1\|AHSG\|ALB\|APOA1\|FN1\|HRG\|ITGA2B\|ORM1\|ORM2\|SERPINA4\|SERPINF2\|THBS1\|VCL\|TAGLN2 | 36 | 24 | -17 |
| GO MF | GO:0005539 | glycosaminoglycan binding | BGN\|FN1\|CFH\|HRG\|IGHM\|JCHAIN\|LPA\|SERPINA5\|SAA1\|SELL\|SOD3\|THBS1\|THBS4\|TNXB\|NRP1 | 19 | 16 | -12 |
| GO CC | GO:0034358 | plasma lipoprotein particle | APOA1\|APOA4\|APOC3\|CETP\|HPR\|LPA\|PLTP\|SAA1\|PCYOX1 | 73 | 25 | -12 |
| GO BP | GO:0043062 | extracellular structure organization | A2M\|ALB\|APOA1\|APOA4\|APOC3\|BGN\|CETP\|COL6A3\|CPB2\|FN1\|GSN\|ITGA2B\|LPA\|MMP9\|SERPINF2\|PLTP\|THBS1\|TNXB | 12 | 14 | -11 |
| GO BP | GO:0009611 | response to wounding | A2M\|ACTN1\|APOA1\|C4BPB\|CPB2\|F12\|FN1\|GSN\|HRG\|ITGA2B\|KRT1\|MCAM\|SERPINA5\|SERPINF2\|SAA1\|THBS1\|VCL\|YWHAZ\|NRP1\|PROZ\|PROCR | 8.8 | 12 | -11 |
| GO BP | GO:0008228 | opsonization | C4BPA\|C4BPB\|CRP\|FCN2\|MBL2 | 120 | 24 | -7 |
| KEGG Pathway | hsa05150 | Staphylococcus aureus infection | C1QA\|C1QB\|C1QC\|C1S\|CFH\|KRT10\|MBL2 | 36 | 16 | -6.6 |
| GO BP | GO:0018149 | peptide cross-linking | BGN\|FN1\|KRT1\|KRT2\|KRT10\|THBS1 | 51 | 17 | -6.4 |
| GO BP | GO:0010955 | negative regulation of protein processing | A2M\|C4BPA\|C4BPB\|CPB2\|SERPINF2\|THBS1 | 43 | 16 | -5.9 |
| GO CC | GO:0071753 | IgM immunoglobulin complex | IGHM\|JCHAIN\|IGKV3-20 | 290 | 29 | -5.1 |
| GO MF | GO:0004252 | serine-type endopeptidase activity | C1S\|CD5L\|F12\|HP\|HPR\|LPA\|MMP9\|PROZ | 14 | 9.9 | -4.7 |
| GO MF | GO:0005509 | calcium ion binding | ACTN1\|C1S\|CDH13\|CRP\|MEGF8\|F12\|GSN\|SELL\|THBS1\|THBS4\|AOC3\|PROZ\|CD93\|CRTAC1 | 5.8 | 7.6 | -4.6 |
| GO BP | GO:0051702 | interaction with symbiont | CRP\|FN1\|CFHR1\|HRG\|MBL2\|DCD | 19 | 10 | -4 |
| GO BP | GO:0007044 | cell-substrate junction assembly | ACTN1\|FN1\|HRG\|THBS1\|VCL\|NRP1 | 18 | 9.7 | -3.7 |
| GO MF | GO:0050839 | cell adhesion molecule binding | ACTN1\|CDH13\|ENO1\|FN1\|SELL\|THBS1\|THBS4\|TNXB\|VCL\|YWHAZ\|TAGLN2 | 6.3 | 7.1 | -3.7 |
| GO BP | GO:0050704 | regulation of interleukin-1 secretion | APOA1\|IGHD\|ORM1\|ORM2\|SAA1 | 25 | 11 | -3.7 |
