# Supplementary material for: A Novel Diagnostic Predictive Model for Idiopathic Short Stature in Children
Source: Front Endocrinol (Lausanne). 2021 Sep 17;12:721812. doi: 10.3389/fendo.2021.721812 (PMC8485046; doi:10.3389/fendo.2021.721812)
Supplement: Supplementary file 3 [file Table_3.docx]

| **Only Hub Genes** | **Only Top DEPs** | **Hub Genes AND Top DEPs** |
| --- | --- | --- |
| C1QC | JCHAIN | C1QA |
| CRP | BGN | C1QB |
| MBL2 | CD5L |  |
| C1S | IGFBP2 |  |
| KRT10 | SOD3 |  |
| VCL | SAA1 |  |
| CFH | IGHM |  |
| YWHAZ | F12 |  |
| FCN2 |  |  |
| KRT1 |  |  |
| KRT2 |  |  |
| FN1 |  |  |
| GSN |  |  |

**Supplementary Table 3.** The common genes between the top 10 FC DEPs and hub genes from MCODE networks.
