# Supplementary material for: A Novel Diagnostic Predictive Model for Idiopathic Short Stature in Children
Source: Front Endocrinol (Lausanne). 2021 Sep 17;12:721812. doi: 10.3389/fendo.2021.721812 (PMC8485046; doi:10.3389/fendo.2021.721812)
Supplement: Supplementary file 4 [file Table_4.docx]

**Supplementary Table S4. Patients information**

| No. | Group | Gender | Height,cm | Age | Chronological Age | Weight | WBC | CRP | C1QA | C1QB |
| --- | --- | --- | --- | --- | --- | --- | --- | --- | --- | --- |
| 1 | ISS1 | Male | 122.3 | 10 | 7 | 23 | 6.14 | 12.27 | - | - |
| 2 | ISS2 | Male | 124 | 11 | 9 | 19 | 9.52 | 4.52 | - | - |
| 3 | ISS3 | Female | 129.5 | 10.4 | 7 | 24 | 10.7 | 6.31 | - | - |
| 4 | ISS4 | Female | 116 | 8 | 5 | 17 | 6.02 | 5.87 | - | - |
| 5 | NC1 | Female | 133.2 | 8.6 | 8 | 23 | 6.21 | 1.06 | - | - |
| 6 | NC2 | Male | 143 | 10 | 9 | 31 | 7.36 | 1.15 | - | - |
| 7 | NC3 | Male | 146 | 11 | 11 | 42 | 5.94 | 0.32 | - | - |
| 8 | NC4 | Female | 142.3 | 10.8 | 9 | 29 | 6.21 | 0.14 | - | - |
| 9 | ISS | Male | 96.3 | 5.2 | 4 | 15.2 | 6.66 | 7.81 | 926.908 | 1488.549 |
| 10 | ISS | Female | 90.4 | 5.9 | 4 | 18 | 7.91 | 6.12 | 815.071 | 1474.985 |
| 11 | ISS | Female | 94.9 | 5.5 | 4 | 14.3 | 6.76 | 5.87 | 1520.106 | 1190.639 |
| 12 | ISS | Female | 109.2 | 7 | 5 | 19.2 | 7.68 | 4.44 | 646.7174 | 1196.738 |
| 13 | ISS | Male | 118.2 | 7.4 | 5 | 23.1 | 7.48 | 4.35 | 780.1235 | 1125.964 |
| 14 | ISS | Female | 116.3 | 8 | 5 | 17.8 | 6.02 | 5.61 | 1057.614 | 864.8031 |
| 15 | ISS | Female | 111.6 | 8.5 | 5 | 18.5 | 8.87 | 6.12 | 722.5019 | 1012.589 |
| 16 | ISS | Male | 122 | 9.6 | 7 | 21.2 | 9.26 | 3.45 | 761.0979 | 1018.089 |
| 17 | ISS | Female | 113 | 9 | 7 | 22.3 | 6.22 | 4.12 | 1414.099 | 1548.118 |
| 18 | ISS | Female | 135 | 9.8 | 6 | 31.2 | 5.84 | 6.87 | 1291.848 | 1175.49 |
| 19 | ISS | Female | 121.3 | 9 | 8 | 19.5 | 5.73 | 6.12 | 765.9865 | 1206.954 |
| 20 | ISS | Male | 119 | 9.2 | 7 | 21.3 | 6.19 | 12.78 | 941.9191 | 1387.035 |
| 21 | ISS | Female | 127.1 | 10 | 7 | 23 | 6.14 | 10.11 | 785.689 | 873.288 |
| 22 | ISS | Male | 119.9 | 10.4 | 8 | 25.6 | 10.7 | 9.31 | 1137.463 | 836.2243 |
| 23 | ISS | Male | 127 | 10.3 | 7 | 24.3 | 7.36 | 11.11 | 1373.355 | 1353.994 |
| 24 | ISS | Male | 126.5 | 10.7 | 5 | 24.1 | 7.9 | 12.27 | 762.9294 | 975.4373 |
| 25 | ISS | Male | 124 | 11 | 6 | 23.6 | 9.52 | 8.21 | 985.062 | 1133.791 |
| 26 | ISS | Female | 144 | 12.4 | 6 | 34.5 | 5.8 | 0.42 | 822.6605 | 1494.76 |
| 27 | ISS | Female | 138 | 12 | 7 | 38.6 | 7.61 | 3.21 | 1455.542 | 1089.264 |
| 28 | ISS | Male | 133.5 | 12.9 | 10 | 25.4 | 14.43 | 3.12 | 1133.509 | 1000.727 |
| 29 | ISS | Male | 139 | 12.4 | 9 | 37.6 | 6.45 | 2.32 | 1178.525 | 1051.449 |
| 30 | ISS | Male | 141.6 | 13.6 | 8 | 38.3 | 5.82 | 0.21 | 1175.255 | 1579.628 |
| 31 | ISS | Male | 140.1 | 13 | 10 | 37.3 | 7.89 | 4.12 | 777.656 | 1517.363 |
| 32 | ISS | Male | 143 | 13.4 | 11 | 37.2 | 3.98 | 1.21 | 1257.196 | 1028.217 |
| 33 | ISS | Male | 85.5 | 5.8 | 3 | 13.2 | 7.78 | 8.57 | 1023.773 | 1078.784 |
| 34 | ISS | Female | 89.2 | 5 | 3 | 14.6 | 9.03 | 6.88 | 1131.142 | 916.2754 |
| 35 | ISS | Female | 102.7 | 5.5 | 3 | 13.6 | 7.88 | 6.63 | 857.9235 | 1345.008 |
| 36 | ISS | Female | 108.3 | 7 | 4 | 18.2 | 8.8 | 5.2 | 998.5722 | 2046.921 |
| 37 | ISS | Male | 117 | 7.6 | 4 | 22 | 8.6 | 5.11 | 1367.527 | 1741.648 |
| 38 | ISS | Female | 115 | 8.5 | 4 | 16.2 | 7.14 | 6.37 | 978.0295 | 942.5369 |
| 39 | ISS | Female | 110.4 | 8 | 4 | 16.8 | 9.99 | 6.88 | 633.6478 | 885.2288 |
| 40 | ISS | Male | 121 | 9 | 6 | 19.7 | 10.38 | 4.21 | 720.1191 | 1044.914 |
| 41 | ISS | Female | 122.3 | 9.7 | 6 | 19.2 | 7.34 | 4.88 | 1086.227 | 812.1676 |
| 42 | ISS | Female | 126 | 9.4 | 5 | 30 | 6.96 | 7.63 | 964.0334 | 740.0637 |
| 43 | ISS | Female | 120.9 | 9 | 7 | 18.3 | 6.85 | 6.88 | 848.8714 | 1156.498 |
| 44 | ISS | Male | 118 | 9 | 6 | 19.9 | 7.31 | 13.54 | 1174.439 | 937.2555 |
| 45 | ISS | Female | 126.3 | 10.5 | 6 | 22.1 | 7.26 | 10.87 | 956.3934 | 1021.766 |
| 46 | ISS | Male | 123.6 | 10 | 7 | 24.3 | 11.82 | 10.07 | 1167.929 | 1267.518 |
| 47 | ISS | Male | 126.4 | 10.3 | 6 | 23.7 | 8.48 | 11.87 | 1101.539 | 1629.82 |
| 48 | ISS | Male | 124.9 | 10 | 4 | 23.5 | 9.02 | 13.03 | 1435.635 | 1866.322 |
| 49 | ISS | Male | 127 | 11.5 | 5 | 22.2 | 10.64 | 8.97 | 926.908 | 1488.549 |
| 50 | ISS | Female | 136 | 12 | 5 | 33.1 | 6.92 | 0.13 | 815.071 | 1474.985 |
| 51 | ISS | Female | 137 | 12.7 | 6 | 37.5 | 8.73 | 3.97 | 920.1064 | 1190.639 |
| 52 | ISS | Male | 132.4 | 12.3 | 9 | 23.7 | 15.55 | 3.88 | 746.7174 | 1196.738 |
| 53 | ISS | Male | 138.6 | 12.5 | 8 | 35.8 | 7.57 | 3.08 | 780.1235 | 1125.964 |
| 54 | ISS | Male | 143.1 | 13 | 7 | 36.8 | 6.94 | 0.43 | 1057.614 | 864.8031 |
| 55 | ISS | Male | 142 | 13.9 | 9 | 38.7 | 6.77 | 2.63 | 722.5019 | 1012.589 |
| 56 | ISS | Male | 148.7 | 14 | 8 | 40.2 | 6.14 | 0.31 | 761.0979 | 1018.089 |
| 57 | ISS | Male | 149 | 14.2 | 10 | 39.1 | 8.21 | 4.43 | 1414.099 | 1548.118 |
| 58 | ISS | Male | 150.2 | 14.6 | 11 | 39.1 | 4.3 | 1.52 | 1291.848 | 1175.49 |
| 59 | ISS | Male | 104.5 | 6 | 3 | 15.6 | 8.1 | 8.88 | 765.9865 | 1206.954 |
| 60 | ISS | Female | 106.2 | 6.5 | 3 | 18.2 | 9.35 | 7.19 | 941.9191 | 1387.035 |
| 61 | ISS | Female | 108.3 | 6.9 | 3 | 15.7 | 8.2 | 6.94 | 785.689 | 873.288 |
| 62 | ISS | Female | 111.9 | 8 | 4 | 20.1 | 9.12 | 5.51 | 1137.463 | 836.2243 |
| 63 | ISS | Male | 120 | 8.3 | 4 | 24.1 | 8.92 | 5.42 | 1373.355 | 1353.994 |
| 64 | ISS | Female | 118.6 | 9 | 4 | 18.2 | 7.46 | 6.68 | 762.9294 | 975.4373 |
| 65 | ISS | Female | 117.5 | 9.7 | 4 | 19.1 | 10.31 | 7.19 | 1585.062 | 1133.791 |
| 66 | ISS | Male | 124.8 | 10 | 6 | 21.8 | 10.7 | 4.52 | 822.6605 | 1494.76 |
| 67 | ISS | Female | 122.4 | 10.5 | 6 | 21.9 | 7.66 | 5.19 | 1455.542 | 1089.264 |
| 68 | ISS | Female | 128 | 10 | 5 | 32.1 | 7.28 | 7.94 | 1133.509 | 1000.727 |
| 69 | ISS | Female | 123.9 | 10.6 | 7 | 20.2 | 7.17 | 7.19 | 1178.525 | 1051.449 |
| 70 | ISS | Male | 125.4 | 10 | 6 | 22 | 7.63 | 13.85 | 1175.255 | 1579.628 |
| 71 | ISS | Female | 129 | 11.7 | 6 | 24 | 7.58 | 11.18 | 777.656 | 1517.363 |
| 72 | ISS | Male | 123.9 | 11 | 7 | 25.9 | 12.14 | 10.38 | 1257.196 | 1028.217 |
| 73 | ISS | Male | 129.8 | 11.4 | 6 | 24.9 | 8.8 | 12.18 | 1023.773 | 1078.784 |
| 74 | ISS | Male | 126.7 | 11 | 4 | 25 | 9.34 | 13.34 | 1131.142 | 916.2754 |
| 75 | ISS | Male | 130 | 12.7 | 5 | 24.3 | 10.96 | 9.28 | 857.9235 | 1345.008 |
| 76 | ISS | Female | 141 | 13 | 5 | 35.1 | 7.24 | 0.44 | 998.5722 | 2046.921 |
| 77 | ISS | Female | 140.3 | 13.8 | 6 | 38.9 | 9.05 | 4.28 | 1367.527 | 1741.648 |
| 78 | ISS | Male | 92.4 | 5 | 4 | 15.7 | 6.01 | 7.35 | 978.0295 | 942.5369 |
| 79 | ISS | Female | 101.4 | 5.5 | 4 | 21.0 | 7.26 | 5.66 | 633.6478 | 885.2288 |
| 80 | ISS | Female | 97.2 | 5 | 4 | 16.2 | 6.11 | 5.41 | 720.1191 | 1044.914 |
| 81 | ISS | Female | 116.9 | 7.8 | 5 | 20.9 | 7.03 | 3.98 | 1086.227 | 812.1676 |
| 82 | ISS | Male | 117.1 | 7 | 5 | 24.8 | 6.83 | 3.89 | 964.0334 | 740.0637 |
| 83 | ISS | Female | 119.9 | 8.3 | 5 | 18.7 | 5.37 | 5.15 | 848.8714 | 1156.498 |
| 84 | ISS | Female | 124.5 | 8 | 5 | 20.3 | 8.22 | 5.66 | 1174.439 | 937.2555 |
| 85 | ISS | Male | 125 | 9.6 | 7 | 23 | 8.61 | 2.99 | 956.3934 | 1021.766 |
| 86 | ISS | Female | 126.9 | 9.9 | 7 | 23.2 | 5.57 | 3.66 | 1167.929 | 1267.518 |
| 87 | ISS | Female | 121 | 9.5 | 6 | 33 | 5.19 | 6.41 | 1101.539 | 1629.82 |
| 88 | ISS | Female | 124.8 | 9 | 8 | 21 | 5.08 | 5.66 | 1435.635 | 1866.322 |
| 89 | NC | Female | 119 | 5.2 | 5 | 26.3 | 5.73 | 0.21 | 892.0212 | 832.056 |
| 90 | NC | Male | 119.2 | 7 | 7 | 23.4 | 9.96 | 0.65 | 1106.168 | 617.0802 |
| 91 | NC | Male | 124 | 8.6 | 8 | 29.3 | 6.52 | 0.34 | 508.4398 | 826.2348 |
| 92 | NC | Female | 125.8 | 8 | 8 | 31.9 | 8.97 | 1.06 | 1082.426 | 910.1993 |
| 93 | NC | Male | 130.9 | 9.6 | 9 | 32.7 | 5.9 | 0.31 | 878.159 | 828.7275 |
| 94 | NC | Male | 132.1 | 9 | 9 | 27.4 | 10.94 | 0.25 | 723.0981 | 852.144 |
| 95 | NC | Female | 134 | 10.8 | 10 | 31.2 | 9.64 | 0.48 | 579.5605 | 938.1347 |
| 96 | NC | Female | 139 | 10.7 | 9 | 40.5 | 11.24 | 0.29 | 769.6626 | 748.8403 |
| 97 | NC | Male | 136.4 | 10 | 10 | 39.2 | 8.12 | 0.37 | 485.8422 | 610.3224 |
| 98 | NC | Female | 136 | 10.5 | 11 | 28 | 6.21 | 0.26 | 836.6702 | 863.1106 |
| 99 | NC | Female | 137.2 | 10 | 10 | 27.5 | 8.31 | 0.19 | 780.7409 | 812.1676 |
| 100 | NC | Female | 144.5 | 12.7 | 12 | 36.5 | 9.49 | 0.54 | 683.0259 | 854.6694 |
| 101 | NC | Male | 159 | 14 | 15 | 52.4 | 4.81 | 0.78 | 943.978 | 517.782 |
| 102 | NC | Female | 118 | 7.5 | 7 | 27.8 | 5 | 0.23 | 942.6051 | 863.1106 |
| 103 | NC | Male | 117.8 | 6.1 | 6 | 24.9 | 6.15 | 0.95 | 810.0314 | 664.2065 |
| 104 | NC | Male | 129 | 8.8 | 8 | 30 | 4.73 | 0.2 | 729.6707 | 1030.989 |
| 105 | NC | Female | 122.5 | 8.4 | 8 | 33.2 | 5 | 0.14 | 744.7055 | 690.4899 |
| 106 | NC | Male | 132 | 9.7 | 9 | 34 | 5.45 | 0.37 | 661.0348 | 739.2676 |
| 107 | NC | Male | 127 | 9.6 | 9 | 28.2 | 6.7 | 0.18 | 785.0697 | 778.6368 |
| 108 | NC | Female | 135.8 | 10 | 10 | 32.3 | 5.55 | 0.26 | 726.6799 | 832.056 |
| 109 | NC | Female | 137 | 10.8 | 9 | 41.2 | 6.47 | 0.15 | 741.0847 | 1068.366 |
| 110 | NC | Male | 136.9 | 10.3 | 10 | 40 | 6.27 | 0.08 | 559.8913 | 859.7297 |
| 111 | NC | Female | 135 | 10.4 | 11 | 29.1 | 4.81 | 0.43 | 798.1261 | 1095.966 |
| 112 | NC | Female | 120 | 7.8 | 7 | 28.2 | 7.66 | 0.26 | 988.6177 | 950.4868 |
| 113 | NC | Male | 119.7 | 7 | 8 | 25.1 | 8.05 | 0.19 | 665.0843 | 790.8403 |
| 114 | NC | Male | 125 | 8.5 | 8 | 31.2 | 5.01 | 0.32 | 899.3335 | 941.6557 |
| 115 | NC | Female | 127.6 | 8.7 | 9 | 34.1 | 4.63 | 0.37 | 876.189 | 922.3708 |
| 116 | NC | Male | 130 | 9 | 9 | 35.3 | 5.65 | 0.3 | 690.6141 | 847.103 |
| 117 | NC | Male | 133.2 | 9.4 | 10 | 29 | 5.27 | 0.44 | 545.8155 | 899.8276 |
| 118 | NC | Female | 135 | 10 | 9 | 33 | 5.16 | 0.48 | 715.9572 | 1120.117 |
| 119 | NC | Female | 136 | 10.5 | 10 | 42.1 | 5.62 | 1.21 | 820.7597 | 890.3688 |
| 120 | NC | Male | 135.4 | 10 | 11 | 41.3 | 8.68 | 2.45 | 582.8594 | 1119.144 |
| 121 | NC | Female | 140 | 10.4 | 7 | 30.1 | 10.3 | 6.32 | 420.101 | 1952.929 |
| 122 | NC | Female | 123 | 7 | 8 | 29.1 | 6.58 | 0.47 | 481.1462 | 1377.846 |
| 123 | NC | Male | 123.9 | 7.4 | 8 | 26.3 | 8.39 | 0.87 | 862.4695 | 1366.439 |
| 124 | NC | Male | 128 | 8 | 9 | 32.1 | 15.21 | 3.54 | 973.8163 | 933.7427 |
| 125 | NC | Female | 128.5 | 8.2 | 9 | 34.9 | 7.23 | 0.67 | 871.6021 | 762.4774 |
| 126 | NC | Male | 133 | 9.6 | 10 | 35.8 | 6.6 | 0.29 | 958.473 | 478.5274 |
| 127 | NC | Female | 147.1 | 12 | 12 | 39.7 | 6.43 | 0.37 | 642.3721 | 478.5274 |
| 128 | NC | Male | 157.8 | 13.9 | 14 | 57 | 5.8 | 0.26 | 466.1906 | 1106.551 |
| 129 | NC | Female | 127 | 7.5 | 7 | 32.4 | 7.87 | 0.19 | 892.0212 | 832.056 |
| 130 | NC | Male | 127.8 | 7.6 | 7 | 29.3 | 3.96 | 0.21 | 706.1685 | 617.0802 |
| 131 | NC | Male | 132 | 8.4 | 8 | 34 | 7.76 | 0.78 | 508.4398 | 826.2348 |
| 132 | NC | Female | 133.4 | 8.9 | 8 | 37 | 9.01 | 1.01 | 882.4265 | 910.1993 |
| 133 | NC | Male | 137 | 9 | 9 | 38 | 7.86 | 1.06 | 878.159 | 828.7275 |
| 134 | NC | Male | 136.9 | 9.6 | 9 | 32.1 | 8.78 | 0.31 | 723.0981 | 852.144 |
| 135 | NC | Female | 142 | 10 | 10 | 36.1 | 8.58 | 0.25 | 579.5605 | 938.1347 |
| 136 | NC | Female | 148 | 12.5 | 12 | 42.1 | 7.12 | 0.48 | 769.6626 | 748.8403 |
| 137 | NC | Male | 153.5 | 14 | 14 | 58.2 | 9.97 | 0.29 | 485.8422 | 610.3224 |
| 138 | NC | Female | 144 | 12.7 | 12 | 35.9 | 10.36 | 0.37 | 836.6702 | 863.1106 |
| 139 | NC | Male | 157.6 | 14 | 15 | 53.2 | 7.32 | 0.26 | 780.7409 | 812.1676 |
| 140 | NC | Female | 149.3 | 12.6 | 12 | 44.2 | 6.94 | 0.19 | 683.0259 | 854.6694 |
| 141 | NC | Male | 158 | 14.3 | 14 | 58.9 | 6.83 | 0.54 | 843.978 | 517.782 |
| 142 | NC | Female | 145 | 12 | 12 | 37.2 | 7.29 | 0.78 | 942.6051 | 863.1106 |
| 143 | NC | Male | 155.8 | 14.1 | 15 | 52.9 | 7.24 | 0.23 | 810.0314 | 664.2065 |
| 144 | NC | Female | 117 | 7.3 | 7 | 27.3 | 11.8 | 3.21 | 729.6707 | 1030.989 |
| 145 | NC | Male | 116.8 | 7.7 | 7 | 24.1 | 8.46 | 0.2 | 744.7055 | 690.4899 |
| 146 | NC | Male | 122 | 8.4 | 8 | 29.9 | 9 | 0.14 | 661.0348 | 739.2676 |
| 147 | NC | Female | 124.8 | 8 | 8 | 32.8 | 10.62 | 2.34 | 785.0697 | 778.6368 |
| 148 | NC | Male | 127 | 9.7 | 9 | 33.9 | 6.9 | 0.18 | 726.6799 | 832.056 |
| 149 | NC | Male | 129.5 | 9 | 9 | 27.8 | 8.71 | 1.32 | 741.0847 | 1068.366 |
| 150 | NC | Female | 132 | 10.6 | 10 | 31.9 | 5.67 | 0.67 | 559.8913 | 859.7297 |
| 151 | NC | Female | 137.8 | 10 | 9 | 41.2 | 6.92 | 0.29 | 798.1261 | 1095.966 |
| 152 | NC | Male | 138.6 | 10.2 | 10 | 40 | 5.77 | 0.37 | 988.6177 | 950.4868 |
| 153 | NC | Female | 137 | 10.6 | 11 | 29.1 | 6.69 | 0.26 | 765.0843 | 790.8403 |
| 154 | NC | Female | 124.6 | 7 | 7 | 29.2 | 6.49 | 0.19 | 899.3335 | 941.6557 |
| 155 | NC | Male | 120 | 7.4 | 8 | 25.1 | 5.03 | 0.21 | 876.189 | 922.3708 |
| 156 | NC | Male | 125.6 | 8 | 8 | 31.2 | 7.88 | 0.78 | 690.6141 | 847.103 |
| 157 | NC | Female | 127 | 8.6 | 9 | 34.3 | 8.27 | 0.31 | 545.8155 | 899.8276 |
| 158 | NC | Male | 130.7 | 9 | 9 | 34.8 | 5.23 | 0.25 | 715.9572 | 1120.117 |
| 159 | NC | Male | 130 | 9.6 | 10 | 29.2 | 4.85 | 0.48 | 820.7597 | 890.3688 |
| 160 | NC | Female | 135.6 | 10 | 9 | 32.9 | 4.74 | 0.29 | 582.8594 | 1119.144 |
| 161 | NC | Female | 137.8 | 10.4 | 10 | 41.8 | 5.39 | 0.37 | 420.101 | 1952.929 |
| 162 | NC | Female | 137 | 10.6 | 7 | 30.4 | 9.62 | 0.26 | 481.1462 | 1377.846 |
| 163 | NC | Female | 121.9 | 7 | 8 | 29.1 | 6.18 | 0.19 | 862.4695 | 1366.439 |
| 164 | NC | Male | 123 | 7.4 | 8 | 26.2 | 8.63 | 0.54 | 973.8163 | 933.7427 |
| 165 | NC | Male | 126.7 | 8 | 9 | 32.1 | 5.56 | 0.78 | 871.6021 | 762.4774 |
| 166 | NC | Female | 125.9 | 8.7 | 9 | 34.8 | 10.6 | 3.21 | 958.473 | 478.5274 |
| 167 | NC | Male | 133 | 9.6 | 10 | 36.2 | 9.3 | 1.99 | 742.3721 | 478.5274 |
| 168 | NC | Female | 144.8 | 12.6 | 12 | 39.9 | 10.9 | 2.39 | 666.1906 | 1106.551 |
